# Резолюция по итогам междисциплинарного экспертного совета «Профилактика и лечение ожирения. Как достичь здорового метаболического баланса»

**DOI:** 10.14341/probl13211

**Published:** 2022-12-20

**Authors:** Е. А. Трошина, Л. А. Суплотова, Т. Л. Каронова, Ф. Х. Дзгоева, О. В. Васюкова, О. В. Ремизов, И. Б. Туаева, Е. Л. Хадарцева, Л. А. Руяткина, З. Р. Гусова, Е. В. Екушева, Н. Г. Мокрышева

**Affiliations:** Национальный медицинский исследовательский центр эндокринологии; Тюменский государственный медицинский университет; Национальный медицинский исследовательский центр им. В.А. Алмазова; Национальный медицинский исследовательский центр эндокринологии; Национальный медицинский исследовательский центр эндокринологии; Северо-Осетинская государственная медицинская академия; Северо-Кавказский многопрофильный медицинский центр; Северо-Кавказский многопрофильный медицинский центр; Новосибирский государственный медицинский университет; Ростовский государственный медицинский университет; Федеральный научно-клинический центр специализированных видов медицинской помощи и медицинских технологий ФМБА России; Национальный медицинский исследовательский центр эндокринологии

**Keywords:** ожирение, коморбидность, профилактика, лечение, ВОЗ, сердечно-сосудистые заболевания, СД 2 типа

## Abstract

30 сентября 2022 г. во Владикавказе состоялось заседание междисциплинарного экспертного совета «Профилактика и лечение ожирения. Как достичь здорового метаболического баланса». Для снижения социального и экономического бремени ожирения и его последствий в Российской Федерации необходимо внедрять социально значимые инициативы по профилактике ожирения и повышению его выявляемости, а также актуализировать современные подходы к лечению этого хронического заболевания с учетом его многофакторного патогенеза, коморбидного фона, риска развития осложнений и инвалидизации пациентов. По результатам проведенных в ходе экспертного совета научных докладов и дискуссии эксперты приняли решения по дальнейшему плану в рамках социально значимых инициатив по профилактике ожирения.

## Abstract (en)

On September 30, 2022, a meeting of the interdisciplinary expert council “Prevention and treatment of obesity. How to Achieve a Healthy Metabolic Balance. To reduce the social and economic burden of obesity and its consequences in the Russian Federation, it is necessary to introduce socially significant initiatives to prevent obesity and increase its detection rate, as well as to update modern approaches to the treatment of this chronic disease, taking into account its multifactorial pathogenesis, comorbidity, risk of complications and patient disability. Based on the results of the scientific reports and discussions held during the expert council, the experts made decisions on a further plan within the framework of socially significant initiatives for the prevention of obesity

Проблема избыточной массы тела и ожирения в Российской Федерации приобрела масштабы пандемии. По последним данным, около 65% трудоспособного населения страны имеют лишний вес, включая ожирение разной степени выраженности. Отмечаются также высокие темпы роста ожирения и избыточного веса у детей и подростков. Социальная значимость данной проблемы определяется угрозой дезадаптации, инвалидизации пациентов молодого и среднего возраста и снижением продолжительности жизни в связи с частым развитием сопутствующих заболеваний, влиянием ожирения на метаболическое и ментальное здоровье современного человека.

Доказано, что ожирение является самостоятельным фактором риска развития сердечно-сосудистых и цереброваскулярных (в том числе инсульта) заболеваний, инсульта, сахарного диабета 2 типа и других заболеваний, многие из которых известны как «болезни старения цивилизации». По данным Всемирной организации здравоохранения (ВОЗ), 44% случаев развития сахарного диабета, 23% случаев ишемической болезни сердца и 80% случаев ряда онкологических заболеваний обусловлены избыточной массой тела и ожирением. Предшествующие сердечно-сосудистые события, а также хроническое системное воспаление, возникающее и персистирующее на фоне избытка жировой ткани, лежат в основе патологических изменений головного мозга. Так, последние данные системных обзоров и метаанализов показали, что риск возникновения ишемического инсульта и деменции у пациентов с ожирением увеличивается более чем в 2 раза. В целом ожирение значимо влияет на качество жизни и уменьшает ее продолжительность в среднем на 3–5 лет при небольшом избытке веса и до 15 лет при выраженном ожирении.

В современном обществе массовый характер приобрели аддиктивные нарушения, в том числе связанные с нарушением пищевого поведения. Данные нарушения отличаются устойчивыми тенденциями к росту и представляют собой серьезную медицинскую, социальную, педагогическую и психологическую проблему. В связи с чем ВОЗ предлагает объединить и усилить меры по охране ментального здоровья как неотъемлемого компонента здоровья, качества жизни и благополучия человека. При этом ВОЗ отмечает, что ожирение является высококоморбидным заболеванием, ассоциированным с высокой частотой развития когнитивных нарушений, включая деменцию.

Для снижения социального и экономического бремени ожирения и его последствий в Российской Федерации необходимо внедрять социально значимые инициативы по профилактике ожирения и повышению его выявляемости, а также актуализировать современные подходы к лечению этого хронического заболевания с учетом его многофакторного патогенеза, коморбидного фона, риска развития осложнений и инвалидизации.

По результатам проведенных в ходе Совещания научных докладов и дискуссии экспертного совета было признано нижеследующее.

По результатам проведенных в ходе Совещания научных докладов и дискуссий экспертным советом было принято решение.

Резолюция одобрена и принята единогласно по результатам открытого голосования участников Экспертного Совета 30.09.2022 г.

## ДОПОЛНИТЕЛЬНАЯ ИНФОРМАЦИЯ

Источник финансирования. Работа выполнена по инициативе авторов без привлечения финансирования.

Конфликт интересов. Авторы декларируют отсутствие явных и потенциальных конфликтов интересов, связанных с публикацией настоящей статьи.

Участие авторов. Все авторы одобрили финальную версию статьи перед публикацией, выразили согласие нести ответственность за все аспекты работы, подразумевающую надлежащее изучение и решение вопросов, связанных с точностью или добросовестностью любой части работы

Благодарности. Выражается благодарность Ванатовой Ольге Владимировне, Исаевой Умулкулсум Солтановне, Темирдашевой Наталье Михайловне, Барахоевой Розе Махмутовне, Славицкой Елене Семеновне, Камалову Камалу Гаджиевичу, Шихалиевой Инне Николаевне, Бабковой Наталье Валериевне.

